# Molecular detection of equid herpesvirus in bronchoalveolar lavage fluid from asymptomatic horses in Southern Brazil

**DOI:** 10.14202/vetworld.2022.2597-2602

**Published:** 2022-11-17

**Authors:** Mariane Angélica Finger, Janaina Socolovski Biava, Peterson Triches Dornbusch, João Henrique Perotta, Leila Sabrina Ullmann, Priscila Beatriz da SiIva Serpa, Louise Bach Kmetiuk, Andrea Pires dos Santos, Alexander Welker Biondo, Christian Leutenegger, Ivan Roque de Barros Filho

**Affiliations:** 1 Department of Veterinary Medicine, UFPR - Federal University of Paraná, Rua dos Funcionários, 1540, Curitiba, PR, 80035, Brazil; 2 Department of Animal Science, College of Agriculture “Luiz de Queiroz”, University of São Paulo, Piracicaba, SP, 13418-900, Brazil; 3 Biotechnology Institute, Sao Paulo State University (UNESP), Botucatu, 18607-440, SP, Brazil; 4 Department of Biomedical Sciences and Pathobiology, Virginia-Maryland College of Veterinary Medicine. Virginia Tech, Blacksburg, VA, 24061, USA; 5 Department of Comparative Pathobiology, Purdue University, West Lafayette, IN, 47907, USA; 6 Director Molecular Diagnostics, Antech Diagnostics - Molecular Diagnostics, Fountain Valley, CA, 92708, USA

**Keywords:** bronchoalveolar lavage fluid, endoscopy, equine, gammaherpesvirus, polymerase chain reaction, tracheal wash

## Abstract

**Background and Aim:**

Molecular approaches to diagnose respiratory viruses have provided an opportunity for early and subclinical pathogen detection, particularly in samples from the upper respiratory tract. This study aimed to investigate the presence of herpesviruses, particularly equid herpesvirus (EHV)-2 and EHV-5, in samples from the lower respiratory tract of healthy racehorses from Southern Brazil.

**Materials and Methods:**

Samples from the lower respiratory tract (i.e., bronchoalveolar lavage fluid [BALF]) were assessed by video endoscopy, cytological evaluation of BALF, and tracheal aspirates (TA), along with quantitative polymerase chain reaction (qPCR), to detect equine herpesvirus infection in the lower respiratory tract samples and compare corresponding cytological and endoscopic findings.

**Results:**

At least one abnormality per horse during endoscopy examination was observed, including, but not limited to, mucous secretion in the airways and pharyngeal lymphoid hyperplasia. The presence of EHV-2 and/or EHV-5 was detected by qPCR in 3/10 animals. One horse was positive for EHV-2 alone, one for EHV-5 alone, and one for both.

**Conclusion:**

To the authors’ knowledge, this is the first molecular detection of EHV-2 and EHV-5 in Brazilian thoroughbred horses. These findings may provide new insights into the epidemiology of EHV-2 and EHV-5 in Brazilian horses, evidencing the importance of the molecular investigation, early detection, and prevention of respiratory diseases.

## Introduction

Respiratory diseases are a well-known cause of poor athletic performance in horses. Different methods have been applied for early and precise diagnosis of respiratory diseases after the recognition of poor performance, such as airway endoscopy, cytology, and assessment of lung function [1]. The ability to control or minimize the effects of some of these diseases depends on the diagnosis of possible etiological agents, particularly viruses. Molecular biology techniques have been widely used to detect active infections or to identify carriers of some important agents, such as equine influenza virus (EIV), equine rhinovirus, and equid herpesvirus (EHV) [2].

Five out of nine identified species of equid herpesviruses infect the domestic horse. Two belong to the subfamily gammaherpesvirinae (EHV-2 and EHV-5) and three to the subfamily alphaherpesvirinae (EHV-1, EHV-3, and EHV-4) [3, 4]. Except EHV-3, a venereal pathogen, all herpesviruses cause upper or lower respiratory diseases in horses and are endemic worldwide [5]. EHV-2 and EHV-5 have variable presentations, from pharyngitis, lymphadenomegaly, fever, and anorexia to pneumonia or multinodular pulmonary fibrosis (EMPF) [6]. However, like other herpesviruses, the infection can be asymptomatic, latent, or act as a co-factor for the development of other diseases, and the full pathogenic potential of gammaherpesviruses remains unclear [5, 6].

In Brazil, the first isolation of equid herpesvirus was described in 1966. However, there have been few cases and investigations on the current prevalence of gammaherpesvirus in Brazil, with one case report of EMPF [7] and one recent evaluation in two farms that detected EHV-2 and EHV-5 in samples from the upper airway secretions of asymptomatic horses [8].

Thus, this study aimed to investigate the presence of herpesviruses, especially EHV-2 and EHV-5, in samples from the lower respiratory tract (i.e., bronchoalveolar lavage fluid and BALF) and evaluate whether the infection with these viruses causes alterations in the respiratory tract of healthy athletic horses from Southern Brazil, assessed by videoendoscopy, complete blood count (CBC), and cytological evaluation of BALF and tracheal aspirates (TA).

## Materials and Methods

### Ethical approval

This study was approved by the Ethics Committee in Animal Use (protocol number: 042/2012) at the Federal University of Parana.

### Study period and location

The study was conducted from January 2014 to January 2015 at State Jockey Club, Curitiba, Southern Brazil. The study included ten standard bred young horses with ages ranging from 2 to 3 years (mean: 2.14 ± 0.06), namely, eight males and two females. To determine the healthy condition by standard physical examination, the horses were housed in the veterinary facility State Jockey Club, Brazil, and determined to be healthy by standard physical examination. Horses were housed in individual stables with a sawdust bed, and daily fed with approximately 6 kg of whole oat groats and 2 kg of commercial concentrate (Supra Alisul Foods, São Paulo, Brazil), in addition to hay and water *ad libitum*.

### Blood sampling

Approximately 10 mL of whole blood was collected from the jugular vein of each horse and immediately transferred to ethylenediaminetetraacetic acid tubes for CBC. After sampling, horses were transferred back to their individual stables. Blood samples were stored on ice packs (4°C), shipped to a clinical pathology laboratory, and analyzed within 24 h of collection. The CBC was performed in an automated cell counter (BC-2800Vet, Mindray Bio-Medical Electronics Co. Shenzhen Shi, China), followed by a manual differential count. Total plasma protein and fibrinogen were determined by refractometry, using the heat-precipitation method.

### Videoendoscopic examination

The animals underwent 1 h of training, approximately 24 h before blood collection, videoendoscopy, and related procedures. After intravenous sedation (1 mg/kg xylazine, Rompun, Bayer Animal Health, Leverkusen, Germany), a flexible videoendoscope (Karl Storz, Endoscopy-America, Inc, ElSegundo, CA, USA) was passed through the right nostril and advanced along the ventral meatus, caudally to the nasopharynx. Structural and functional aspects of the nasal cavity, nasopharynx, guttural pouches, and larynx were examined, including the presence or absence of secretion, pharyngeal lymphoid hyperplasia (PLH), dorsal displacement of the soft palate (DDSP), epiglottic entrapment (EE), and recurrent laryngeal neuropathy (RLN). The endoscope was advanced caudally into the trachea to the level of the carina. At that time, the presence and amount of mucus and blood in the trachea were assessed. An adapted grading score to assess the quantity and quality of secretion present in the trachea from 0 to 4 was used, where 0 represents the absence of mucus; 1 (small) represents singular threads or droplets of mucus; 2 (larger) represents confluent droplets of mucus; 3 represents streams of mucus within the trachea; and 4 (large) represents streams or pools of mucus that covered more than 25% of the tracheal circumference, as previously described [8, 9].

### Tracheal aspirate technique

At the end of the examination described above, a single-lumen polypropylene catheter was advanced through the endoscope biopsy channel until the catheter tip was positioned immediately proximal to the tracheal bifurcation. The tracheal fluid was aspirated and processed in the laboratory within 30 min of collection. Two evaluators counted at least 400 cells in cytocentrifuge (CT14 model, Teklab, Piracicaba, SP, Brazil) preparations (1488× *g* for 10 min) stained with May-Grünwald Giemsa (MGG).

### Bronchoalveolar lavage fluid collection technique

Briefly, a bronchoalveolar lavage tube (11 mm × 245 cm VBAL30, Bivona, Smiths Medical, Dublin, OH, USA) was passed blindly into the distal airway. After the infusion of 20 mL of 2% lidocaine (Bravet, Laboratory Bravet, Rio de Janeiro, Brazil) to desensitize the airways, the tube was gently advanced into the bronchial tree. Two 50 mL aliquots of sterile 0.9% saline were infused into the alveolar space and gently aspirated. The average proportion of recovered fluid was 36.5% of the original volume infused. All BALF samples were kept on ice and processed within 12 h of collection. Cytocentrifuge preparations were prepared and evaluated as the TA samples. Approximately 1 mL of BALF was frozen at −20°C at the time of sampling and sent to the IDEXX Laboratories (Sacramento, CA, USA) on ice packs within 24–48 h of collection.

### Polymerase chain reaction (PCR)

Quantitative PCR (qPCR, Comprehensive Equine Respiratory RealPCR Panel, IDEXX Laboratories, Inc., Westbrook, ME, USA) was used for the detection of EHV-2 and EHV-5. The test also enables the detection of equine adenovirus (EAdV), EIV (H3N8), EHV-1, EHV-4, and equine rhinovirus types A (ERAV) and B (ERBV), in addition to *Streptococcus equi* subsp. equi, *Streptococcus dysgalactiae* subsp. equisimilis, and *Streptococcus equi* subsp. zooepidemicus. All assays were designed and validated in accordance with industry standards, and details are of proprietary rights, including diagnostic sensitivity and specificity and the lowest number of DNA copies of each pathogen that can be detected.

### Statistical analysis

All data were tested for a normal distribution using the D’Agostino and Pearson omnibus test. Animals positive and negative for gammaherpesvirus were compared using Mann–Whitney’s test or unpaired t-test with Welch’s correction. The null hypothesis was that the distributions or means were identical between the two groups. Linear logistic regression was performed to calculate the probability of predicting positivity for gammaherpesvirus based on the percentage of cells present in both BALF and TA. The correlation between videoendoscopic findings and positivity for gammaherpesvirus using Spearman’s test was also determined. Alpha was set at 0.05 (GraphPad Prism 8.0.2, GraphPad, San Diego, CA, USA).

## Results

Ten healthy thoroughbred horses (2–3 years old) under regular training and housed at the Paraná State Jockey Club, Brazil were evaluated. The CBC data of the horses are presented in Table-1. No horse was positive for EAdV, EIV/H3N8, EHV-1, EHV-4, ERAV, ERBV, or *Streptococcus* spp. However, one horse was positive for EHV-2 (Horse 4), one for EHV-5 (Horse 10), and one for both EHV-2 and EHV-5 (Horse 6) (Table-2). The comparison between animals positive and negative for herpesvirus showed no significant difference in any hematological variable between horses positive or negative for gammaherpesvirus.

**Table-1:** Hematologic parameters in young healthy horses at training (mean ± standard deviation).

Hematologic parameters	Horses (n = 10)	Reference values
RBC (×10^6^/µL)	11.2 ± 0.6	6.6–9.7
Hematocrit (%)	44.4 ± 1.8	34–46
Hemoglobin (g/dL)	15.3 ± 1.0	11.8–15.9
Platelets (×10^3^/µL)	194.0 ± 122.9	94–232
WBC (×10^3^/µL)	7.7 ± 1.0	5.2–10.1
Neuts (×10^3^/µL)	49.2 ± 7.8	2.7–6.6
Bands (×10^3^/µL)	0.7 ± 0.9	0
Lymphs (×10^3^/µL)	48.2 ± 8.4	1.2–4.9
Eos (×10^3^/µL)	1.1 ± 1.1	0–1.2
Monos (×10^3^/µL)	0.6 ± 0.9	0–0.6
Basos (×10^3^/µL)	0.1 ± 0.3	0–0.2
Total protein	6.6 ± 0.3	5.2–7.8
Fibrinogen-heat precipitation (mg/dL)	260.0 ± 126.4	0–200

RBC=Red blood cell count, WBC=White blood cell count

**Table-2:** Respiratory fluid evaluation in young healthy horses (n = 10) at training (mean ± standard deviation) for tracheal aspirate and BALF.

Differential cells count (mL) (%)	Tracheal aspirate	BALF
Total cells		
Macrophage	175.7 ± 67.7 (43.9 ± 16.9%)	252.9 ± 43.6 (63.2 ± 10.9)
Lymphocytes	94.1 ± 48.0 (23.5 ± 12.0%)	90.7 ± 22.6 (22.6 ± 5.6%)
Neutrophils	81.1 ± 87.6 (20.2 ± 21.9)	44.4 ± 39.7 (11.1 ± 9.9%)
Eosinophils	3.5 ± 4.4 (0.8 ± 1.1%)	3.6 ± 5.1 (0.9 ± 1.2%)
Mast cells	3.6 ± 3.3 (0.9 ± 0.8%)	5.7 ± 5.2 (1.4 ± 1.3%)
Epithelial cells	45.4 ± 59.9 (10.9 ± 15.2%)	7.1 ± 4.5 (1.7 ± 1.1%)
Goblet cells	3.9 ± 10.2 (0.1 ± 2.5)	n.d. (n.d.)
Hemosiderophages	n.d. (n.d.)	n.d. (6.6 ± 7.4%)
THSs	n.d. (n.d.)	9.7 ± 11.7 (n.d.)

BALF=Bronchoalveolar lavage fluid, THSs=Mean total hemosiderin scores, n.d.=Not determined. *Normal ranges are indicated in brackets

Animal number six had mild evidence of an inflammatory process as revealed by an increase in the number of bands representing 28 × 10^9^/L. There were two animals with mild thrombocytosis (Horses 5 and 8, 385 and 355 × 10^9^/L, respectively) and two with marked thrombocytopenia (Horses 3 and 6, 30.5 and 26.5 × 10^9^/L, respectively). There were no notable findings regarding the remaining parameters.

Abnormalities observed in the videoendoscopic evaluation are presented in Table-2. Epistaxis, DDSP, and EE were not present in any horse, while PLH was present in all animals. Whenever secretion was observed in the nasal cavities, guttural pouches, pharynx, and trachea, it was mucoid and clear. All horses had mucous secretions in the trachea to different degrees and one animal had a swollen carina. Nasal, pharyngeal, guttural pouch secretions, and left RLN were observed in a few animals (Table-2). There were no correlations among any videoendoscopic findings and none of these findings was predictive of the status of gammaherpesvirus infection (positive or negative test result).

On BALF evaluation (Table-3), only one animal (Horse 5) had normal differential counts (i.e., macrophages 60–80%, lymphocytes 20–35%, neutrophils <5%, mast cells <2%, and eosinophils <1%) [10, 11]. Nine horses had mildly to moderately increased neutrophils. Three horses that had mastocytosis also had an increased proportion of neutrophils. Considering the TA evaluation (Table-2), no animal had a normal differential count (namely, macrophages 40%-80%, neutrophils <20%, lymphocytes <10%, mast cells <1%, and eosinophils <1%) [10, 11], although three animals (Horses 1, 2, and 5) had only mildly increased numbers of lymphocytes. Seven horses had more neutrophils in their TA than in their BALF. However, only two horses reached the cutoff for neutrophilic inflammation (>20%) [11]; these two animals were positive for herpesvirus. One horse had lymphocytic inflammation (lymphocytes were the predominant cell type). There was no relationship between the proportion of cells in both TA and BALF and the probability of testing positive for herpesvirus. However, the proportion of neutrophils in TA showed a tendency for significance regarding an association with the probability of testing positive for herpesvirus (p = 0.0527).

**Table-3:** Detection of EHV-1, EHV-2, EHV-4, EHV-5, equine rhinovirus A, equine rhinovirus, equine adenovirus-1 and B, *S. equi* in BALF from young healthy horses at training in Brazil (n = 10) based on virus-specific qPCR.

qPCR testing	Horse									

	1	2	3	4	5	6	7	8	9	10
EIV RealPCR	N	N	N	N	N	N	N	N	N	N
EHV-1 neuropath	N	N	N	N	N	N	N	N	N	N
EHV-1 non-neuropath	N	N	N	N	N	N	N	N	N	N
EHV-4 RealPCR	N	N	N	N	N	N	N	N	N	N
*S. equi* PCR	N	N	N	N	N	N	N	N	N	N
Equine rhinovirus A	N	N	N	N	N	N	N	N	N	N
Equine rhinovirus B	N	N	N	N	N	N	N	N	N	N
EHV type 2 RealPCR	N	N	N	N	N	P	N	N	N	N
EHV type 5 RealPCR	N	N	N	P	N	P	N	N	N	P
Equine adenovirus-1	N	N	N	N	N	N	N	N	N	N

qPCR=Quantitative polymerase chain reaction, EIV RealPCR=Equine influenza virus, EHV-1 Neuropath=Equine herpesvirus neuropathogenic, EHV-1 Non-neuropath=Equine herpesvirus no neuropathogenic, EHV-4 RealPCR=Equine herpesvirus type 4, *S. equi* PCR=*Streptococcus* *equi* subsp. *equi*, Equine rhinovirus A=Equine rhinitis A vírus, Equine rhinovirus B=Equine rhinitis B virus, EHV type 2 RealPCR=Equine herpesvirus type 2, EHV type 5 RealPCR=Equine herpesvirus type 5, Equine adenovirus-1=Equine adenovirus type 1, N=Sample was PCR negative, P=Sample was PCR positive

## Discussion

We described the presence of EHV-2 and EHV-5 detected by qPCR in samples from the lower respiratory tract (i.e., BALF) of clinically healthy, asymptomatic, thoroughbred racehorses, housed at the Paraná State Jockey Club, in Southern Brazil. A recent study detected positivity for gammaherpesvirus in 14 out of 18 sampled Appaloosa horses [8]. Nine animals were positive for EHV-5, four for EHV-2, and one for both. Although there were no details about the animals’ age, athletic condition, and the proportion of animals sampled from the entire farm, the animals were said to be asymptomatic adults [8]. Given this proof that the virus is circulating in Brazil, and more specifically in Paraná State, we considered it vital to evaluate the possible presence of EHV-2 and EHV-5 at the Jockey Club. Animals at this club are housed at a high density and occasionally travel interstate, increasing the risk of contamination and spread of the disease. A recent study analyzed the same infectious agents evaluated herein in owned horses imported into the United States. Among the nasal swabs of 167 animals tested using PCR during a 20-month period, 48 were positive for EHV-2, 68 for EHV-5, and 27 were positive for both gammaherpesvirus [12]. A total of 72 animals were asymptomatic and 44 had various symptoms (e.g., fever, tachypnea, cough, lymphadenopathy, and nasal discharge) [12]. The results emphasized the frequent and silent presence of gammaherpesvirus in equine populations.

In our study, the animal positive for EHV-2 only (Horse 4) had an unremarkable CBC and BALF, but the most severe neutrophilic inflammation in the TA among all animals. However, he only had a small amount of tracheal secretion (Score 1) and bilateral mucus in both guttural pouches, indicating an unremarkable inflammatory reaction in the respiratory tract. In contrast, the animal positive for EHV-5 only (Horse 10) had mild to moderate increases in the number of neutrophils in BALF and TA, respectively, but the marked presence of tracheal mucus (Score 4), in addition to nasal and pharyngeal secretion. The single double-positive horse (Horse 6) had neutrophilic inflammation in its BALF, but unremarkable TA, and only a small amount of tracheal secretion. Interestingly, this was the animal with severe thrombocytopenia and a mild inflammatory leukogram. These inconsistent videoendoscopic and cytological findings highlight the lack of correlation and predictive value of these evaluations in identifying gammaherpesvirus infection, emphasizing the importance of molecular diagnostics.

The most consistent videoendoscopic finding in all animals was PLH, which is significantly higher in younger, asymptomatic racehorses than in older ones. Despite its high prevalence in thoroughbreds (63%), PLH does not appear to affect performance [13–15]. A second consistent finding was tracheal secretion to various degrees in all horses. Although it may be a sign of inflammatory airway disease (IAD), it is insufficient to reach a diagnosis since none of the horses had any history of chronic cough or poor performance. In addition, considering the higher cutoffs for BALF differential counts currently proposed for IAD diagnosis (>10% neutrophils, >5% eosinophils, and >5% mast cells) [1], this disease was not present in our study population.

Although not significantly correlated with gammaherpesvirus infection, the presence of neutrophils in TA appeared to be the closest indicator of possible presence of the virus in the respiratory tract. However, athletic horses can experience transient increases in cells as an adaptation to intensive training, which has been reported to alter the lung’s innate immune response and systemic circulation [16]. The overall poor correlation between TA and BALF in our study has previously been described, indicating the distinct responses of tracheal and bronchioalveolar epithelia to stimuli. In addition, the trachea is the natural site from which inflammatory debris exits the lower bronchial tree [13–15]. Therefore, an inflammatory process may have been resolved in the lower respiratory tree and be currently “passing through” the trachea. Besides, both tissues may be concurrently affected, or infections can progress from the upper to the lower tract.

The role gammaherpesviruses play in the development of respiratory disease remains poorly understood. However, molecular diagnostic techniques such as qPCR have been pivotal tools for the early and rapid detection of infectious diseases [5]. Nonetheless, several epidemiological investigations on the prevalence of herpesvirus may have been affected by the type of sample utilized. For instance, whole blood, peripheral blood mononuclear cells, conjunctival swabs, nasal swabs, and lower respiratory secretions (i.e., TA and BALF, as in our study) have been described, the selection of which can potentially affect the sensitivity of the test [15, 17, 18]. To our knowledge, test sensitivity for each sample type has not been determined to date.

Moreover, the higher prevalence of gammaherpesvirus compared with that of alphaherpesvirus found by others and us [19, 20] is likely due to the lack of availability of vaccines for EHV-2 and EHV-5, although experimental immunization of foals against EHV-2 has been reported [21].

Regardless of the study’s small sample size, we demonstrated 30% prevalence of gammaherpesvirus in horses with no pathognomonic clinical signs or relevant hematological, videoendoscopic, and cytological findings. None of the parameters evaluated would reliably predict such viral infection.

## Conclusion

The detection of gammaherpesvirus in places with a high transit of animals, such as Paraná State Jockey Club in our study, reinforces the value of molecular techniques for identifying potential viral reservoirs and disseminators. Investigations of asymptomatic or subclinical conditions in racehorses can potentially minimize the considerable costs associated with poor performance linked to these viruses.

## Data Availability

The datasets generated during and/or analyzed during the current study are available from the corresponding author on reasonable request.

## Authors’ Contributions

MAF, JSB, PTD, JHP, AWB, and IRBF: Data collection. MAF, JSB, PTD, JHP, AWB, and IRBF: Data analysis. MAF, JSB, PTD, JHP, LSU, PBSS, LBK, APS, AWB, CL, and IRBF: Study design and interpretation of the data. MAF, LBK, APS, AWB, and IRBF: Drafted and revised the manuscript. All authors have read and approved the final manuscript.
